# Capecitabine-induced severe diabetes and hypokalemia: a case report

**DOI:** 10.1186/s13256-022-03392-w

**Published:** 2022-04-25

**Authors:** Qiaoling Yang, Chuping Chen, Jianmin Ran

**Affiliations:** 1 Guizhou Medical University, Guizhou, 550025 China; 2 Endocrinology Department, Guangzhou Red Cross Hospital, No. 396, Tong Fu Zhong Road, Guangzhou, 510220 China

**Keywords:** Breast cancer, Capecitabine, 5-Fluorouracil, Diabetes, Hypokalemia, Case report

## Abstract

**Background:**

Capecitabine is widely used in chemotherapy for breast, colorectal, and gastric cancers. The frequent adverse reactions of capecitabine mainly include gastrointestinal side effects, anemia, and cardiovascular toxicity. Here, we report a rare case of severe hyperglycemia and hypokalemia during long-term treatment with capecitabine.

**Case presentation:**

A 48-year-old Chinese female was hospitalized with the complaint of breathlessness and weakness after activity, for 1 month. Her past history is significant for a diagnosis of right-sided breast cancer 7 years ago. She underwent right mastectomy, following which capecitabine was started 1.5 years prior to the current admission as part of her primary treatment at the discovery of systemic osseous metastasis. Her fasting plasma glucose and hemoglobin A1c levels were quite normal 7 months ago but increased to 15.3 mmol/L and 11.2%, respectively, at the present admission. Her serum potassium level was as low as 2.5 mmol/L. Plasma autoantibodies related to islets and insulin were all negative. Capecitabine was discontinued, and an insulin pump and potassium supplement were given after admission. Her blood sugar and potassium levels returned to their normal ranges soon. Self-injection of insulin was withdrawn completely at 2 months after discharge, and no oral hypoglycemic agents were added. Her plasma glucose and electrolyte levels were at normal levels at her 1-year follow-up.

**Conclusion:**

Glucose intolerance and hypokalemia may be rare but serious adverse effects during long-term chemotherapy with capecitabine.

## Background

Capecitabine is widely used as monotherapy or combination therapy for chemotherapy of breast, colorectal, and gastric cancers. The frequent adverse reactions of this agent mainly include gastrointestinal side effects, anemia, cardiovascular toxicity, central nervous system damage, and hand–foot syndrome [1]. Here, we report a rare case of severe hyperglycemia and hypokalemia during long-term chemotherapy with capecitabine and complete remission after its discontinuation.

## Case presentation

A 48-year-old Chinese female was hospitalized with the complaint of breathlessness and weakness after activity, for 1 month. The patient was diagnosed with papillary thyroid cancer (PTC) and right breast cancer simultaneously 7 years prior. Bilateral extended thyroidectomy was first performed, and PTC was confirmed by pathology. She then developed hypothyroidism and routinely took l-thyroxine 100 μg/day as replacement therapy. Three months after thyroidectomy, she underwent modified radical mastectomy of the right breast with lymph node dissection. The pathological results were categorized as stage IIIB (pT4N0M0) according to the AJCC 8th edition standards. The immunochemical staining showed ER (+++), PR (+++), and HER-2 (−), and the fraction of Ki-67-positive cells was nearly 30%. After surgery, she was successively administered chemotherapy with an epirubicin + cyclophosphamide (EC) plan, irradiation in DT4480cGY, and endocrine therapy with tamoxifen or goserelin. Zoledronic acid had been regularly injected every 6 months for 5 years. Local ultrasound and serum tumor markers on follow-up remained in the normal range during this period. Unfortunately, systemic osseous metastasis was found 1.5 years before current admission by PET-CT inspection, and capecitabine was then added at a dosage of 1250 mg/m^2^/day twice daily for 2 weeks followed by 1-week cessation. Simultaneously, intermittent endocrine therapy and zoledronic acid injection were administered. Laboratory reports from 7 months prior to this admission showed that her fasting plasma glucose (FPG) level was 5.5 mmol/L and her hemoglobin A1c (HbA1c) was 6.3%. Both values are within the normal range. She did not take any medications other than the above prescriptions in the prior 1.5 years.

**Fig. 1:**
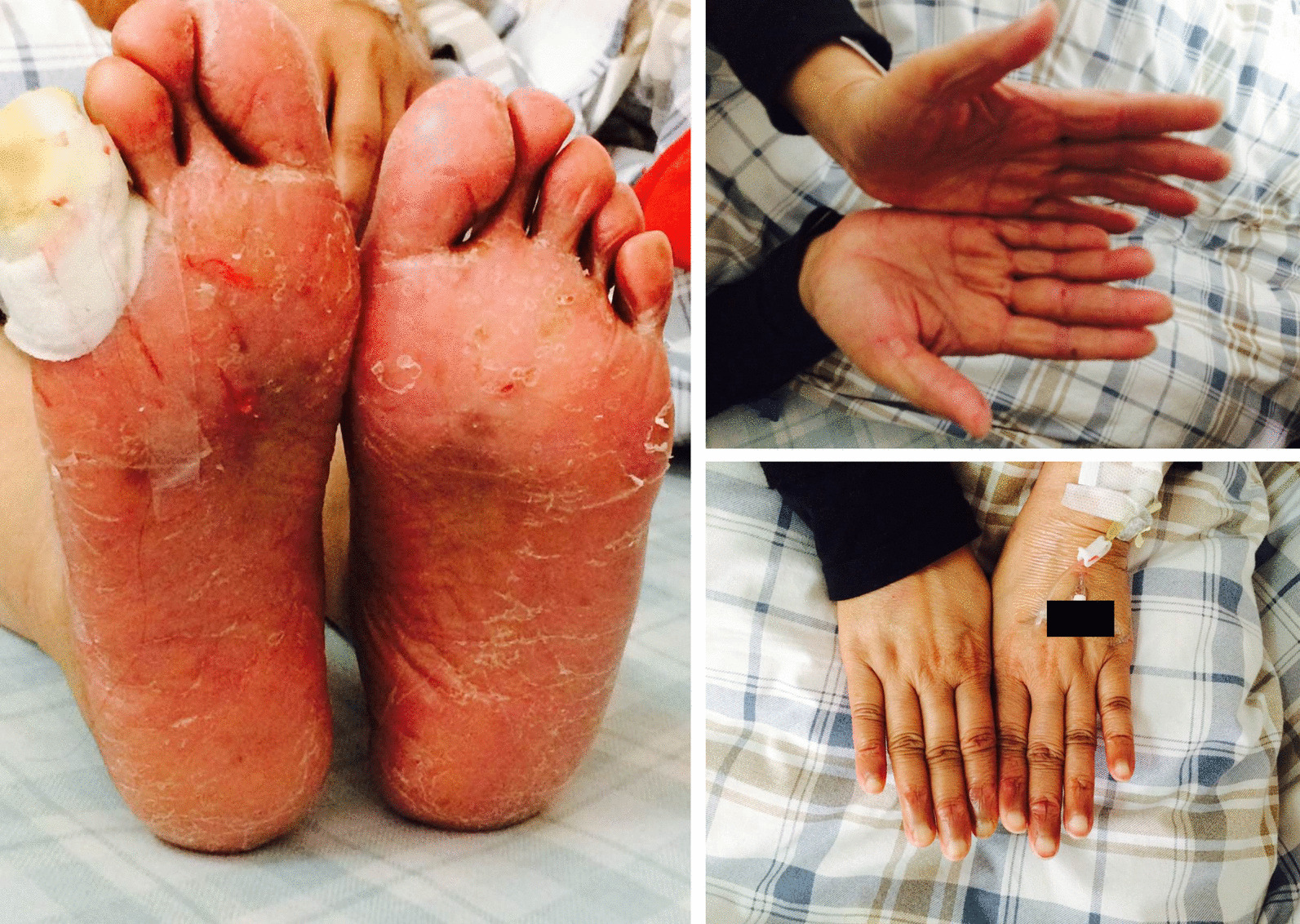
The hands and feet of the patient showed obvious swelling. Diffuse erythema, hyperkeratosis, peeling, fissures with bleeding, and focal erosions were easily seen on both the soul of the foot and toes. The shallow ulcer on the fifth right toe was wetly compressed with 0.1% PVP-1 and wrapped by gauze

Her blood pressure was 126/90 mmHg, and her body mass index (BMI) was 20.12 kg/m^2^ at admission. Palmar–plantar erythrodysesthesia (also termed “hand–foot syndrome”) was obvious (Fig. 1), and we categorized it as grade 3 according to the definition of the National Cancer Institute [2]. Chest radiology and Doppler echocardiography showed normal pulmonary and cardiac function. The plasma level of pro-BNP was also normal (265 pg/mL). Diabetic ketoacidosis was excluded according to normal blood gas analysis and ketone levels.

**Fig. 2:**
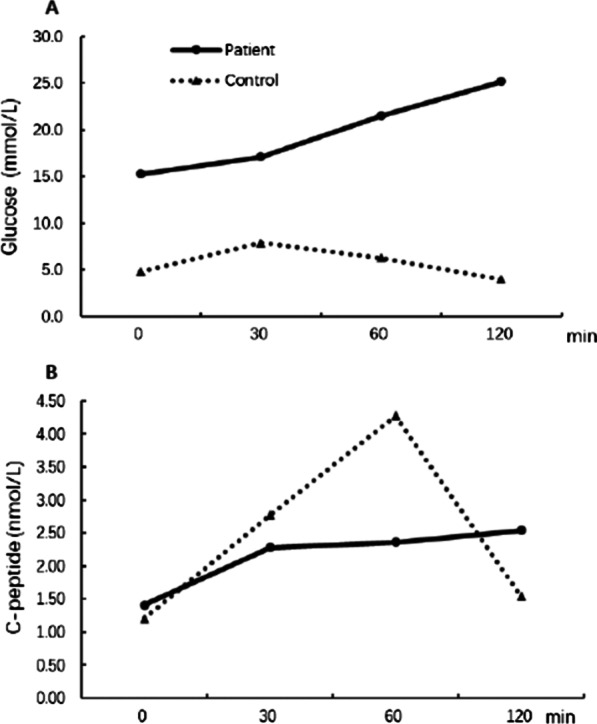
Plasma glucose (**A**) and C-peptide (**B**) profiles from a standard oral glucose tolerance test (OGTT) with challenge of 75 g of glucose. Solid and dashed lines represent the C-peptide profiles of the patient and a healthy control, respectively

The random plasma glucose level (21.7 mmol/L) was significantly increased, and the potassium level (2.5 mmol/L) was decreased. The plasma and urinary ketone determinations were both negative. A standard oral glucose tolerance test (OGTT) with 75 g glucose challenge was performed. As shown in Fig. 2, the patient showed very high levels for FPG (15.3 mmol/L) and 2-h plasma glucose (25.2 mmol/L), confirming the diagnosis of diabetes (panel A). C-peptide was secreted with a normal basal level and a delayed peak concentration compared with a healthy control subject (panel B). The plasma concentration of insulin was not measured since exogenous insulin treatment was initiated immediately after admission. The plasma HbA1c level was 11.2%. All autoantibodies, including islet cell antibody, glutamate decarboxylase antibody, and insulin autoantibody, were negative. The plasma hormones, including thyroid hormones, cortisol, ACTH, aldosterone, angiotensin II, renin activity, gonadal, and gonadotropic hormones, all fell in their normal ranges (data not shown). The daily urinary potassium excretion increased (48.2 mg/day), but other electrolytes were normal.

Capecitabine was discontinued after admission, while l-thyroxine supplementation remained. For hand–foot syndrome, mupirocin ointment was applied, and the shallow wound on the fifth right toe was wetly compressed with 0.1% PVP-1. Subcutaneous insulin pump therapy was applied, and potassium replacement was administered through oral and intravenous approaches. After 1 week of treatment, her FPG fell within 4.0–6.0 mmol/L, and her potassium increased to above 3.5 mmol/L. After discharge, self-injection of premixed insulin and diminishing dose titration continued for approximately 2 months, and was then finally withdrawn. Potassium replacement was completely stopped 1 month after discharge. Plasma glucose and electrolytes levels remained at normal levels during 1-year follow-up. The latest plasma HbA1c was 5.2%, and potassium was 4.1 mmol/L. After discontinuation of capecitabine, endocrine therapy combining goserelin with letrozole was administered, and intermittent injection of zoledronic acid was used as described above. Her symptoms related to bone metastasis, especially ostalgia, were obviously attenuated for the following several months.

## Discussion

Adjuvant addition of capecitabine after neoadjuvant chemotherapy has been confirmed to be safe and effective for prolonging disease-free survival among patients with HER-2-negative breast cancer [3, 4]. Other previous studies have also demonstrated that capecitabine is effective and well tolerated in patients with bone metastases [5]. Recently, the combination of capecitabine with other agents, including endocrine therapies, has been recommended for patients with bone-metastatic breast cancer in the guidelines of the European Society for Medical Oncology (ESMO) [6]. For our patient, we considered capecitabine with endocrine therapy as an optimal selection after bone metastasis, because she had undergone an operation and the following comprehensive medical therapeutics.

Diabetes was definitive in this case, but the reason why diabetes developed is unclear. The patient obtained normal BMI and normal recent FPG and HbA1c levels. She had no family history of diabetes and no tendency for ketoacidosis, and was not positive for autoantibodies. Her insulin secretion presented with abnormal amounts and secretary modes, but most importantly, her diabetes completely reversed after discontinuation of capecitabine treatment. Overall, we may conclude that the diabetes in this patient could not be categorized as classical type 1 or type 2 but might be directly attributed to capecitabine therapy.

Garg *et al.* first reported a case of a patient taking capecitabine who had severe hypertriglyceridemia and diabetes in 2009 [7]. The patient was a 56-year-old male who underwent seven cycles of capecitabine therapy for metastatic colon cancer, then developed typical symptoms of diabetes with significantly increased serum levels of glucose (38.6 mmol/L) and triglyceride (41 mmol/L). Six months after capecitabine discontinuation and specific treatments, hyperglycemia and hypertriglyceridemia were restored to normal state completely. But since then, no other similar cases have been documented. Capecitabine is a prodrug of 5′-deoxy-5-fluorouridine, which is then converted to 5-fluorouracil (5-FU) by both tumor and normal cells [8]. 5-FU has been widely used in colorectal cancer, breast cancer, and other carcinomas [8]. There have been an increasing number of studies showing that 5-FU-based chemotherapy can impair glucose tolerance in patients and even induce severe diabetes with acute complications such as diabetic ketoacidosis [9–11]. In particular, Feng *et al.* [11] found that 11.6% and 11.3% of patients (*n* = 362) with colorectal cancer developed diabetes and impaired fasting glucose after 5-FU therapy, respectively. Thus, the mechanism of capecitabine-induced diabetes may be closely related to 5-FU. Tayek et al. [12] previously found that treatment with 5-FU in patients with colorectal cancer significantly increased fasting hepatic glucose production (HGP) and plasma glucose levels, but the insulin response to intravenous glucose challenge remained unchanged. However, Feng *et al.* [13] explored alterations in insulin secretion and islet structure in Wistar rats treated with 5-FU and verified that 5-FU-induced hyperglycemia is partly attributable to insulin secretion deficiency and islet structural damage. In the present patient, a delayed C-peptide secretary mode indicated impaired insulin secretion, so we speculated that direct islet injury might be an important reason for her diabetes. The diabetes in this patient was completely reversed after discontinuation of the drug, and this phenomenon illustrated that mechanisms other than beta cell function might underlie capecitabine-induced diabetes. Additionally, with reference to the study from Feng *et al.* [11], 16.7% of the cases with diabetes were restored to normal fasting glucose levels without any hypoglycemic intervention. The serum levels of amylase and lipase (data not shown) were quite normal, and acute pancreatitis was excluded. However, pancreatic exocrine injury might serve as a severe complication during capecitabine treatment [14].

Severe hypokalemia is also seldom reported to be associated with capecitabine. A previous study [15] reported a hypokalemia rate of 20.4% after capecitabine treatment, and diarrhea was the leading cause. In another case, Sonnenblick *et al.* [16] found that distal renal tubular acidosis induced by capecitabine may be the underlying cause for hypokalemia. Our patient showed no gastrointestinal side effects, and her acid–base balance was good as well. Since potassium is lost in urine, we tested for the related adrenal cortical hormones and obtained no positive results. Taken together, the mechanism of hypokalemia in this patient was unclear and might have resulted from the tubular toxicity of capecitabine.

## Conclusion

We report a patient with metastatic breast cancer who ultimately presented with severe diabetes and hypokalemia after long-term capecitabine treatment. These adverse effects are rare but severe, reminding clinicians to pay more attention to the related symptoms and laboratory evidence.

## Data Availability

The data used to support the findings of this manuscript are available from the corresponding author upon request.
